# Association of cardiovascular risk factors in different anthropometric parameters in overweight or obese adults

**DOI:** 10.1016/j.ahjo.2026.100823

**Published:** 2026-07-01

**Authors:** Motahareh Hasani, Leila Sheikhi, Soodeh Razeghi Jahromi, Maryam Khazdouz, Saleheh Ahmadzadeh, Zahra Rahimi, Saeed Alinejad, Sahar mohammadipoornami, Mastaneh Rajabian Tabesh, Maryam Abolhasani

**Affiliations:** a Department of Nutritional Sciences, School of Health, Golestan University of Medical Sciences, Gorgan, Iran; b Metabolic Disorders Research Center, Golestan University of Medical Sciences, Gorgan, Golestan Province, Iran; c Department of Nutrition, School of Paramedical, Jundishapur University of Medical Sciences, Ahvaz, Iran; d Department of Clinical Nutrition and Dietetics, Faculty of Nutrition and Food Technology, Shahid Beheshti University of Medical Sciences, Tehran, 19816-19573, Iran; e Department of Nutritional Sciences, School of Health, Iran University of Medical Sciences, Tehran, Iran; f Bachelor's Degree in Nutrition and Diet Therapy, Department of Clinical Nutrition, Shahid Beheshti Medical Sciences University, Tehran, Iran; g Student Research Committee, Faculty of Nutrition and Food Technology, National Nutrition and Food Technology Research Institute, Shahid Beheshti University of Medical Sciences, Tehran, Iran; h Student Research Committee, Faculty of Nutrition and Food Technology, National Nutrition and Food, Technology Research Institute, Shahid Beheshti University of Medical Sciences, Tehran, Iran; i Student Research Committee, Alborz University of Medical Sciences, Karaj, Iran; j Sports Medicine Research Center, Tehran University of Medical Sciences, Tehran, Iran; k Cardiac Primary Prevention Research Center, Cardiovascular Diseases Research Centre, Tehran University of Medical Sciences, Tehran, Iran

**Keywords:** Cardiovascular diseases (CVD), Body mass index (BMI), Insulin resistance, Obesity-related comorbidities

## Abstract

**Background:**

Since overweight and obesity stands as a factor to the development of cardiovascular risk factors and the importance of targeted interventions to address their intertwined impact on public health, this investigation aimed to discover the association between cardiovascular risk factors and anthropometric parameters in adults with overweight or obesity.

**Materials and methods:**

This cross-sectional study evaluated 909 overweight or obese participants from Obesity Clinic and Minimally Invasive Surgery Research Center at Sinai Hospital. The participants were examined for lipid profile, glycemic index, and inflammatory indices. Anthropometric parameters were measured according to the World Health Organization (WHO) protocols. Linear and logistic regression methods were used to examine the relationship between various variables.

**Result:**

In the adjusted model, a significant negative association was observed between the LDL/HDL ratio and BMI (*p* = 0.05), whereas HDL showed a positive association with all anthropometric measures. No significant association was found between WC and the variables in the adjusted model. In the unadjusted model, QUICKI demonstrated significant associations with all anthropometric indices, similar to FBS and ESR. Despite, SBP demonstrated a significant association with WHtR (*p* = 0.04). Furthermore, LDL, insulin and HbA1c showed no significant associations with any of the anthropometric measures in either model (*p* > 0.05).

**Conclusion:**

In conclusion, anthropometric indices may provide useful, though variable, insights into cardiovascular risk factors among overweight and obese individuals. Further research with larger samples and longitudinal design is needed to clarify these associations.

## 1 Introduction

The obesity epidemic is escalating rapidly worldwide, particularly in developing countries [1], [2]. Evidence accumulated over recent decades has shown that individuals with obesity are at increased risk of several chronic diseases and comorbidities, including type 2 diabetes (T2D), hypertension, dyslipidemia, stroke, cancer, and cardiovascular diseases (CVD) [3]. One of the major causes of mortality is CVD [4], [5], [6]. Previous studies and meta-analyses have demonstrated that obesity is strongly associated with increased cardiovascular morbidity and mortality [7], [8]. Therefore, understanding cardiovascular risk factors is essential for developing strategies to prevent CVD and reducing mortality and related underlying diseases [9].

BMI is oftentimes used to obesity diagnosis even so alone, it is not sufficient to assessment the cardio-metabolic risk [10]. According to the literature, total mortality and the risk of non-communicable diseases such as diabetes, cardiovascular disease and cancer enhance with increasing body mass index [11], [12], [13], [14]. Consequently, alternative anthropometric indices such as waist circumference (WC), waist-to-hip ratio (WHR), and waist-to-height ratio (WHtR) have been proposed as additional predictors of cardiovascular risk [15], [16]. WC is useful for accumulation of abdominal fat measure and it is highly correlated with visceral fat [17], while WHtR has been suggested as a simple and effective marker of central obesity and CVD risk prediction [18], [19].

Despite numerous studies investigating obesity-related anthropometric measures, the optimal index for predicting cardiometabolic abnormalities remains controversial, as their predictive performance may vary according to age, sex, ethnicity, and population characteristics [20]. Although the relationship between obesity and cardiovascular risk has been extensively investigated, uncertainty remains regarding the most informative anthropometric index for predicting cardiometabolic abnormalities across different populations and ethnic groups. In particular, limited evidence is available from Middle Eastern populations, including Iranian adults with overweight or obesity. Therefore, the present cross-sectional study aimed to evaluate the association between commonly used anthropometric indices, including BMI, WC, WHR, and WHtR, and cardiovascular risk factors among adults with overweight or obesity.

## 2 Materials and methods

### 2.1 Study population and design

The participants of this study consisted of individuals referred to the Obesity Clinic and Minimally Invasive Surgery Research Center at Sinai Hospital, Tehran, Iran, according to the inclusion and exclusion criteria of the present study between 2017 and 2021. Inclusion criteria was included the presence of overweight or obesity (BMI more than 25 kg/m^2^) and not consuming the drugs such as oral contraceptives or osteoporosis drugs. The presence of any disease, chronic disease, angina pectoris, stroke, transient ischemic attack, heart attack, congenital heart disease, any malignancy, renal failure, and hereditary thrombocytopenia were considered as exclusion criteria.

The sample size was considered 909 people (694 females and 215 males, aged 38.55 ± 10.82 years). The case of all patients was evaluated and analyzed based on specific codes. The individuals responsible for registering information and analyzing the specific details of the participants were completely blinded to the study.

### 2.2 Clinical, biochemical parameters assessment

For all participants, a questionnaire containing personal information and medical history was completed by a trained specialist. The participants were referred to an approved Pathobiology Laboratory to measure cardiovascular disease risk factors such as lipid profile, glycemic index. HOMA-IR was calculated as fasting insulin (μU/mL) × fasting glucose (mg/dL) / 405. QUICKI was calculated as 1 / [log(fasting insulin) + log(fasting glucose)]. Blood pressure was measured twice after 5 min of rest in the seated position using a calibrated sphygmomanometer, and the average value was used in analyses. Written informed consent was obtained from all participants prior to enrollment. The study protocol was approved by the Ethics Committee of Tehran University of Medical Sciences and conducted in accordance with the Declaration of Helsinki.

### 2.3 Anthropometric parameters assessment

According to the World Health Organization (WHO) protocol, anthropometric indices (BMI, Waist WC, WHR, WtHR and HC) were measured and calculated [21]. All anthropometric measurements were obtained twice by trained personnel and the average values were used for analysis to improve reproducibility. For measuring weight, a calibrated digital weight scale (Digital Scales Seca, Japan) was used while participants were wearing light clothing and barefooted. For assessing participants s height, we used a height gauge (Seca 206, China). To calculate body mass index (BMI), weight in kilograms is divided by the square of height in meters (kg/m^2^). The normal range was considered as 19–24.9 kg/m^2^, overweight 25–29.9 kg/m^2^, and obesity grade one 30–24.9 kg/m^2^, obesity grade two 35–39.9 kg/m^2^, and obesity grade three ≥40 kg/m^2^ [22]. WC was measured at the midpoint between the lower margin of the last palpable rib and the top of the iliac crest using a non-elastic measuring tape. Hip circumference (HC) was considered the largest circumference of the buttocks. WHR was calculated by dividing WC by hip circumference.

### 2.4 Statistical analysis

Demographic data are presented as the mean ± standard deviation. To assess the normality of data and homogeneity of variances, the Shapiro-Wilk test and Levene test were used, respectively. Variables with non-normal distributions, including triglycerides and insulin levels, were logarithmically transformed before performing regression analyses. To investigate the association between continuous variables and anthropometric indices, the linear regression method (mixed-effects univariable linear regression models) was used. The association between categorical variables, such as CRP and anthropometric indicators, was evaluated using a logistic regression model. Regression results are presented as regression coefficients with corresponding standard errors, *p*-values, and 95% confidence intervals. STATA version 14 statistical software was used to enter information and analyze data. A p-value of equal or less than 0.05 is considered significant.

## 3 Result

### 3.1 Descriptive characteristics

A total of 909 adults with overweight or obesity participated in this study. The anthropometric indices examined included body mass index (BMI), waist circumference (WC), waist-to-hip ratio (WHR), and waist-to-height ratio (WHtR). Biochemical indicators assessed included triglycerides (TG), high-density lipoprotein (HDL), low-density lipoprotein (LDL), LDL/HDL ratio, HOMA-IR, QUICKI, HbA1c, fasting blood sugar (FBS), insulin, systolic blood pressure (SBP), diastolic blood pressure (DBP), C-reactive protein (CRP), and erythrocyte sedimentation rate (ESR). Their baseline characteristics reported in Table 1. The mean age, weight, BMI, WC, WHR and WHtR were38.5, 97.3, 37.04, 113.9, 0.92 and 0.7, respectively.

**Table 1:** Demographic characteristics of the participants.

Characteristics	Mean ± SD (*n* = 909)
Sex (%female)	76.4%
Age (year)	38.52 ± 10.8

Anthropometric measures	
Weight (kg)	97.33 ± 22.87
BMI (kg/cm^2^)	37.04 ± 7.39
Waist (cm)	113.93 ± 18.41
WHR	0.92 ± 0.06
WHtR	0.7 ± 0.11

Biochemical indicators	
Insulin (U/ml)	29.12 ± 3.49
FBS (mg/dl)	109.88 ± 43.03
HOMA-IR	7.88 ± 3.13
QUIKI	0.28 ± 0.00
HbA1c %	5.69 ± 1.75
TG (mg/dl)	137.7 ± 77.34
HDL (mg/dl)	47.12 ± 12.77
LDL (mg/dl)	117.13 ± 43.98
LDL/HDL ratio	2.81 ± 4.44
ESR (mm/h)	27.35 ± 11.58

BMI, body mass index; WC, waist circumference; WHR, waist-to-hip ratio; WHtR, waist-to-height ratio; TG, triglycerides; HDL, high density lipoprotein; LDL, low density lipoprotein; HOMA-IR, homeostatic model assessment for insulin resistance; QUIKI, quantitative insulin sensitivity check index; FBS, fasting blood sugar; SBP, systolic blood pressure; DBP, diastolic blood pressure; ESR, erythrocyte sedimentation rate.

All values are mean ± standard deviation (SD).

### 3.2 The effect of anthropometric measures on lipid profiles

Multiple linear regression analyses were done to evaluate the association between anthropometric measures and cardiovascular risk factors. Results are shown for both unadjusted (Model 1) and adjusted for age and sex (Model 2). A summary of regression coefficients and *p*-values of lipid profile are shown in Table 2. Based on our analysis, HDL was inversely and significantly associated with all anthropometric measures in adjusted model. Despite, LDL and LDL/HDL ratio were completely unrelated to anthropometric measures. While TG presented a significant association with WC (B = 144.17, SE = 40.35, *p* < 0.001) and WHR (B = 0.27, SE = 0.14, *p* = 0.04) in only unadjusted model. Table 2

**Table 2:** Association between total BMI, WC, WHR and WHtR with lipid profile.

Variables		Total BMI		WC		WHR		WHtR	
		B (SE)	P-value	B (SE)	P-value	B (SE)	P-value	B (SE)	P-value
TG	Model^1^	0.10, (0.34)	0.772	0.27, (0.14)	**0.04**	144.17, (40.35)	**<001**	21.6, (21.83)	0.323
	Model^2^	−8.22, (6.07)	0.176	−7.96, (6.06)	0.19	−7.05, (6.05)	0.244	−8.27, (6.07)	0.173
HDL	Model^1^	−0.64, (0.05)	0.266	−0.43, (0.02)	0.063	−10.59, (6.69)	0.11	−1.32, (3.6)	0.712
	Model^2^	−3.77, (0.98)	**<001**	−3.75, (0.98)	**<001**	−3.72, (0.99)	**<001**	−3.8, (0.98)	**<001**
LDL	Model^1^	0.19, (0.19)	0.325	0.10, (0.08)	0.182	51.18, (23.04)	**0.027**	17.43, (12.41)	0.16
	Model^2^	3.75, (3.44)	0.276	3.76, (3.44)	0.274	4.1, (3.44)	0.23	3.64, (3.44)	0.29
LDL/HDL ratio	Model^1^	−0.14, (0.02)	0.46	0.005, (0.008)	0.54	3.53, (2.33)	0.13	0.34, (1.25)	0.784
	Model^2^	−0.66, (0.34)	**0.05**	−0.64, (0.34)	0.063	−0.62, (0.34)	0.07	−0.65, (0.34)	0.061

BMI, body mass index; WC, waist circumference; WHR, waist-to-hip ratio; WHtR, waist-to-height ratio TG, triglycerides; HDL, high density lipoprotein; LDL, low density lipoprotein; Model^1:^ without adjustment. Model^2^: adjusted for age, sex.

Statistically significant *p*-values are in bold.

### 3.3 The effect of anthropometric measures on glycemic factors

According to glycemic factors, FBS presented a significant association with all anthropometric measures in unadjusted model which not stayed notable in adjusted model. The strongest association was for BMI (B = 38.1, SE = 12.07, *p* = 0.002). HOMA-IR showed positive association with all anthropometric measures which was attenuated in adjusted model. Although, HbA1c, QUICKI and insulin demonstrated no significant association after adjustment. (Table 3)

**Table 3:** Association between total BMI, WC, WHR and WHtR with glycemic factors.

Variables		Total BMI		WC		WHR		WHtR	
		B (SE)	P-value	B (SE)	P-value	B (SE)	P-value	B (SE)	P-value
HOMA-IR	Model^1^	0.04, (0.01)	**0.003**	0.01, (0.006)	**<001**	2.76, (1.64)	0.092	2.46, (0.88)	**<001**
	Model^2^	−0.24, (0.24)	0.314	−0.24, (0.24)	0.314	−0.24, (0.24)	0.312	−0.26, (0.24)	0.272
QUIKI	Model^1^	0.00, (0.00)	**<001**	−8.96, (0.00)	**<001**	−0.17, (0.005)	**<001**	−0.12, (0.002)	**<001**
	Model^2^	−6.18, (0.00)	0.929	−6.94, (0.001)	0.92	0.00, (0.001)	0.88	2.68, (0.001)	0.97
HbA1C	Model^1^	−0.01, (0.01)	0.411	−0.002, (0.003)	0.471	−0.09, (0.92)	0.92	−0.28, (0.49)	0.562
	Model^2^	0.52, (0.13)	0.705	0.55, (0.13)	0.688	0.05, (0.13)	0.68	0.05, (0.13)	0.686
FBS	Model^1^	0.57, (0.19)	**0.003**	0.29, (0.07)	**<001**	44.82, (22.55)	**0.04**	38.1, (12.07)	**0.002**
	Model^2^	−4.5, (3.35)	0.175	−4.5, (3.35)	0.17	−4.5, (3.37)	0.182	−4.86, (3.35)	0.147
Insulin	Model^1^	0.005, (0.01)	0.767	−0.002, (0.01)	0.704	−0.43, (1.83)	0.814	−0.66, (0.98)	0.5
	Model^2^	0.73, (0.27)	0.789	0.06, (0.27)	0.823	0.06, (0.27)	0.821	0.07, (0.27)	0.798

BMI, body mass index; WC, waist circumference; WHR, waist-to-hip ratio; WHtR, waist-to-height ratio; HOMA-IR, homeostatic model assessment for insulin resistance; QUIKI, quantitative insulin sensitivity check index; FBS, fasting blood sugar. Model^1:^ without adjustment. Model^2^: adjusted for age, sex.

Statistically significant p-values are in bold.

### 3.4 The effect of anthropometric measures on blood pressure and ESR

Despite DBP, SBP presented a notable association with WHtR in adjusted model. ESR performed strong association with BMI, WC and WHtR in unadjusted model which did not retain in adjusted model (Table 4).

**Table 4:** Association between total BMI, WC, WHR and WHtR with blood pressure and ESR.

Variables		Total BMI		WC		WHR		WHtR	
		B (SE)	P-value	B (SE)	P-value	B (SE)	P-value	B (SE)	P-value
SBP	Model^1^	<001, (17.93)	0.99	0.47, (0.32)	0.15	−4.24, (9.37)	0.65	10.11, (5.03)	0.45
	Model^2^	0.03, (0.08)	0.68	0.04, (0.03)	0.169	−4.39, (9.4)	0.64	10.18, (5.03)	**0.04**
DBP	Model^1^	0.004, (13.06)	0.49	0.19, (0.24)	0.42	4.96, (6.83)	0.46	5.56, (3.67)	0.13
	Model^2^	−0.25, (0.59)	0.668	0.01, (0.02)	0.46	4.74, (6.85)	0.49	5.59, (3.68)	0.129
ESR	Model^1^	0.10, (0.05)	**0.046**	0.04, (0.02)	**0.04**	8.85, (6.06)	0.144	7.92, (3.25)	**0.015**
	Model^2^	−0.31, (0.90)	0.972	0.001, (0.9)	0.99	0.02, (0.9)	0.975	−0.08, (0.9)	0.93

BMI, body mass index; WC, waist circumference; WHR, waist-to-hip ratio; WHtR, waist-to-height ratio; SBP, systolic blood pressure; DBP, diastolic blood pressure; ESR, erythrocyte sedimentation rate. Model^1:^ without adjustment. Model^2^: adjusted for age, sex.

Statistically significant p-values are in bold.

Table 5 showed that there was a significant association between C-reactive protein (CRP) values and total BMI (*p*-value < 001). The association between anthropometric measurements of waist circumference, WHR, WHtR, and BMI with CRP levels presented in Table5. A significant association between CRP values and BMI was observed. Unlike WC and WHtR, a higher WHR was positively associated with increased levels of CRP.

**Table 5:** Odds ratios and 95% confidence intervals for CRP.

Variables		OR	95%CI	P-value
BMI	Model^1^	1.01	1.00, 1.03	0.043
	Model ^2^	0.99	0.98, 1.01	0.82
WC	Model^1^	1.00	0.99, 1.00	0.74
	Model^2^	0.99	0.98, 1.01	0.78
WHR	Model^1^	1.13	1.01, 1.69	0.022
	Model^2^	0.99	0.99, 1.01	0.80
WHtR	Model^1^	1.04	0.34, 3.16	0.95
	Model^2^	0.99	0.98, 1.01	0.65

BMI, body mass index; WC, waist circumference; WHR, waist-to-hip ratio; WHtR, waist-to-height ratio CRP, C-reaction protein.

Model^1:^ without adjustment.

Model^2^: adjusted for age, sex.

Statistically significant p-values are in bold.

## 4 Discussion

The present study contributes to the existing literature by simultaneously comparing multiple anthropometric indices and their associations with cardiovascular risk factors in an Iranian population with overweight or obesity. According to the findings, higher levels of HDL were strongly associated with a reduced risk of cardiovascular diseases. Although elevated LDL levels were associated with WHR in the crude model, the LDL/HDL ratio showed a significant association with BMI after adjusting for confounding variables.

In terms of glycemic measures, all indicators demonstrated significant associations with anthropometric indices in the crude analysis. However, while diastolic blood pressure showed no significant association with any anthropometric measures, systolic blood pressure was significantly associated with WHtR.

Regarding inflammatory markers, CRP and ESR were only significantly associated in the crude model. Our findings align with previous studies, although some variations are expected due to differences in study design and sample size [23], [24], [25]. While we did not find a significant association between lipid profile and WC in the adjusted model, Moges et al. reported that individuals with high WC had a greater risk of cardiovascular disorders, similar to WHtR [26]. Other studies have shown that HDL and LDL levels in obese or overweight individuals are significantly related to waist circumference [27], [28]. Additionally, an increase in waist-to-hip ratio and BMI has been associated with elevated CRP levels, suggesting a link between central obesity and systemic inflammation [29], [30], [31]. WC has been widely recognized as a simple yet effective index of central obesity [32]. However, previous studies indicated that normal measures of WC did not necessarily cause the improvement in lipid profile, highlighting the limitations of WC as a suitable predictor of dyslipidemia [33]. Another interesting finding was that WC showed statistical power in predicting cardiac-metabolic risk factors. Previous studies that emphasized the role of WHtR over WC and BMI have been conducted among Asian and Caucasian individuals [34], [35]. In contrast, studies on Western population indicated that WC is the most important criterion for obesity when predicting CVD risk factors [36], [37]. Supporting this, a survey in the Spanish Mediterranean population highlighted the impact of BMI as well [38]. In addition, a systematic review and meta-analysis of Caucasian populations revealed that, along with WHtR assessment, the WC is strongly linked to CVD risk factors and thus recommends its use in clinical and research studies [16].

In line with our results, Zhang et al. provided evidence that WHtR was the best predictor of hypertension, while other articles confirmed that WHtR predicts CVD better than WC and BMI [39], [40], [41]. Nayak et al. identified WC and BF% as strong predictors of diabetes among prediabetic individuals [42], [43]. Syauqy et al. demonstrated that high CRP levels were significantly associated with high WC and WHR measures [44]. CRP, a key factor in the pathogenesis of atherosclerosis, is directly linked to increased central adiposity. Higher anthropometric indices reflect greater adiposity, which promotes systemic inflammation via adipocyte-mediated cytokine release, ultimately accelerating cardiovascular disease progression [45]. Regarding BMI, a direct and remarkable correlation was discovered between high levels of BMI and increased TG and LDL/HDL ratio, whereas HDL was inversely associated with BMI, WC, and WHR [46]. Similarly, Oktariza et al. discovered a significant association between FBG and both WHR and BMI [47]. Unlike several previous studies that focused primarily on BMI or WC alone, our study simultaneously evaluated BMI, WC, WHR, and WHtR in relation to multiple cardiometabolic risk factors. The findings suggest that WHtR may have greater utility in predicting blood pressure abnormalities, whereas BMI remained associated with inflammatory and lipid-related indices. Our findings are also supported by Zampetti et al., who demonstrated significant associations between alternative anthropometric indices and blood pressure abnormalities in obese individuals [48].

One of the strengths of this study is the consistency between anthropometric indices and most biomarkers evaluated, indicating good internal validity. Additionally, we examined a large sample of 909 subjects who were not taking medications that could interfere with anthropometric or biological markers – a feature rarely seen in similar studies. Another strength is the inclusion of WHR measurements, which are often lacking in other studies.

However, our study has limitations. First, this was a single-center study conducted in Tehran, Iran, which may limit the generalizability of the findings to other populations and ethnic groups. Second, the gender distribution was imbalanced. Third, the wide age range, including many young subjects, might not fully reflect CVD symptoms. Forth, Selection bias may have occurred because participants were recruited from a specialized obesity clinic, and recall bias related to self-reported medical history cannot be completely excluded. Also, the adjustment for confounding variables, particularly age and sex, may have contributed to differences in our results compared to previous studies. In addition, multiple statistical comparisons were performed without formal correction, which may have increased the likelihood of type I error. Finally, given the cross-sectional design of this study, causality cannot be inferred, and prospective cohort studies are needed to determine whether WHtR is indeed superior to WC and BMI in predicting future cardiovascular risks.

## 5 Conclusion

The present study, conducted in a large sample of overweight and obese individuals, suggests that combining assessments of BMI with WC, WHR, or WHtR can serve as essential tools in clinical practice to better define cardiometabolic risk in people with excess body weight. However, to validate these findings, prospective studies are needed, employing standardized populations and outcomes such as mortality or cardiovascular events, with an accurate assessment of the time-event relationship.

## CRediT authorship contribution statement

**Motahareh Hasani:** Writing – review & editing, Project administration, Methodology, Data curation, Conceptualization. **Leila Sheikhi:** Writing – original draft, Data curation. **Soodeh Razeghi Jahromi:** Methodology. **Maryam Khazdouz:** Project administration. **Saleheh Ahmadzadeh:** Software, Project administration. **Zahra Rahimi:** Software, Data curation. **Saeed Alinejad:** Software. **Mastaneh Rajabian Tabesh:** Resources. **Maryam Abolhasani:** Supervision, Resources, Project administration, Methodology.

## Author agreement

The authors would like to advise that all authors listed have contributed to the work and preparing of manuscript. All authors have agreed to submit the manuscript at *International Journal of Cardiology Cardiovascular Risk and Prevention*.

## Ethical statements

The Tehran University of Medical Sciences' medical ethics committee approved the study protocol and the ethics code IR.TUMS.THC.REC.1400.052 was obtained from the above committee.

## Declaration of competing interest

The authors declare no conflict of interest.
